# Risk factors and treatment outcomes of drug-resistant tuberculosis among patients attending Ndola Teaching Hospital, Zambia: a retrospective case-control study

**DOI:** 10.11604/pamj.2026.53.84.50004

**Published:** 2026-02-13

**Authors:** Ricardo Mckees, Jonathan Gwasupika, Ruth Lindizyani Mfune, Clyde Moono Hakayuwa, Mathias Tembo, Chapline Shike Kalasa, Samuel Mwabafu, Ngula Monde, Imukusi Mutanekelwa, Namaunga Kasumu, Maisa Kasanga, Comfort Asante, Steward Mudenda, Victor Daka

**Affiliations:** 1 Department of Public Health, Copperbelt University, Michael Chilufya Sata School of Medicine, Ndola, Zambia,; 2 Department of Clinical Sciences, National Health Research Training Institute, Ndola, Zambia,; 3 Department of Biomedical Sciences, National Health Research Training Institute, Ndola, Zambia,; 4 Chest Diseases Laboratory, Lusaka, Zambia,; 5 Department of Basic Sciences, Copperbelt University, Michael Chilufya Sata School of Medicine, Ndola, Zambia,; 6 Pathology and Microbiology Laboratory, University Teaching Hospital, Lusaka, Zambia,; 7 Department of Internal Medicine, Ndola Teaching Hospital Postal Agency, Ndola, Zambia,; 8 Department of Pharmacy, University of Zambia, School of Health Sciences, Lusaka, Zambia

**Keywords:** Risk factors, tuberculosis, multidrug-resistant tuberculosis, treatment outcomes, Ndola Teaching Hospital

## Abstract

**Introduction:**

multidrug-resistant tuberculosis (MDR-TB), defined as resistance to isoniazid and rifampicin, has become a major threat to the control of TB across the globe. This study aimed to determine the risk factors and treatment outcomes of Drug-resistant tuberculosis (DR-TB) patients attending Ndola Teaching Hospital (NTH) in Ndola, Zambia.

**Methods:**

we conducted a case-control study among DR-TB patients treated at NTH from 2018 - 2020. Medical records of selected MDR-TB cases and drug susceptible TB controls were included. Treatment outcomes of MDR-TB patients were recorded. The multivariable logistic regression was used to evaluate associations between risk factors and MDR-TB infection. Odds ratios and their 95% confidence intervals were reported.

**Results:**

the mean age for the study was 36.67±9.94, and males were the majority 156 (70.9%). Those with HIV were 123 (55.9%), and contact with DR-TB patients were 61 (27.7%). A total of 110 cases and 110 controls were selected for this study. The multivariable logistic regression model indicated a strong association between a family size of 1-5 and occurrence of MDR-TB (aOR (adjusted odds ratio): 1.44, 95% CI (confidence interval), 0.74-2.79; p=0.028*). There was also a strong association between contact with TB patient and MDR-TB (aOR: 1.67, 95% CI 0.56-4.92, p=0.033*). The analysis indicated that living in the rural area had a negative association with MDR-TB development (aOR: 0.42, 95% CI 0.19-0.91, p=0.025*). There was also a negative association between history of previous treatment and developing MDR-TB (aOR: 0.32, 95% CI 0.17-0.61, p<0.001*), as well as that between contact with MDR-TB patient and MDR-TB development (aOR: 0.50, 95% CI 0.16-1.52, p=0.010*). The study further found that of the 110 MDR-TB patients treated, 73.6% had a successful treatment outcome, and 26.4% had an unsuccessful treatment outcome.

**Conclusion:**

the study found that smaller family size and contact with TB patients were significant risk factors for developing MDR-TB, while living in rural areas, having a history of previous TB treatment, and contact with MDR-TB patients were negatively associated with its occurrence. A notable proportion of unsuccessful treatment outcome of some cases underscores the need for strengthened patient-centered care, adherence support, and equitable access to healthcare across both rural and urban settings.

## Introduction

Tuberculosis (TB) is a communicable disease that is a major cause of ill health and one of the leading causes of death worldwide [1]. Until the coronavirus (COVID-19) pandemic, TB was the leading cause of death from a single infectious agent, ranking above HIV/AIDS [2]. The introduction of the first anti-tuberculosis drugs in the world was in 1943, and since then drug resistance has begun to rise and become a major problem and threat for TB control programs in many countries [3]. According to the Centers for Disease Control (CDC), drug-resistant tuberculosis (DR-TB) disease is caused by TB bacteria that are resistant to at least one of the most effective TB medicines used in treatment regimens. In 2023, an estimated 410 000 people developed multidrug- or rifampicin-resistant tuberculosis (MDR/RR-TB) [1]. The key factors driving the emergence and spread of MDR/RR-TB are poor adherence to standard TB treatment regimens, leading to inadequate drug exposure and the development of resistance, coupled with ongoing transmission of drug-resistant strains through close contact with infectious individuals [4]. There are a total of five countries that accounted for over half of the global number of people estimated to have developed MDR/RR-TB in 2024 including India (27% of global cases), Russia (7.4%), Indonesia (7.4%), China (7.3%) and the Philippines (7.2%) (WHO, 2024). A number of African countries have low rates of MDR-TB largely because of low detection rates and the absence of systematic nationwide surveys to determine the extent of the disease burden [5]. Recent studies continue to highlight the association between HIV infection and multidrug-resistant tuberculosis (MDR-TB) in Mozambique and other African countries. Notable findings include a study in Mozambique, assessing the MDR-TB surveillance system in Maputo City reported an increase in MDR-TB cases, from 943 in 2017 to 1,206 in 2018. While in South Africa: a recent study found that patients co-infected with HIV and MDR-TB had an 8% treatment completion rate and an 18% mortality rate, highlighting the severe impact of co-infection on treatment outcomes [6]. A study on the outcomes of MDR-TB in Zambia from 2012 to 2014 revealed that the survival rate of MDR-TB patients was at 20.2 per 100 person-years of follow-up, making it a serious public health challenge [7]. This study aimed to determine risk factors and treatment outcomes of DR-TB patients treated at Ndola Teaching Hospital (NTH), in order to inform policymakers with recommendations for proper management of the patients.

## Methods

**Study design and setting:** this was a retrospective case-control study of MDR-TB patients treated at NTH from 2018-2020 which aimed at reviewing medical records (patient files) of selected MDR-TB patients and patients with drug susceptible TB, using a structured abstraction tool with questions related to the MDR-TB patients. NTH is a third level hospital with a bed capacity of 800, situated in Ndola. It is the second largest city in Zambia in terms of infrastructural development and third in terms of size and population, with a population of 475, 194, after the capital Lusaka and Kitwe [8]. Additionally, it is the provincial capital of the Copperbelt, and lies about 10 kilometers from the border with DR Congo.

**Study population:** the target population included patients treated for TB and DR-TB. Records from 2018 to 2020 were reviewed, cases and controls were selected in a 1: 1 ratio and controlled for age category and sex. The controls composed of persons diagnosed with drug susceptible TB, while the cases composed of persons with DR-TB, with the inclusion and consideration of the following as possible risk factors for MDR-TB; sex, age, marital status, residence, family size, HIV status, Interruption of TB treatment, history of previous treatment, contact with TB patients, contact with DR-TB patients, sub-optimal dosage and poor drug absorption, incorrect management of individual cases by clinician, use of anti-TB drugs of unproven quality and healthcare worker. Data for all patients diagnosed and reported with drug-resistant tuberculosis (DR-TB) at Ndola Teaching Hospital (NTH), Zambia, between 01/01/2018 and 31/12/2020 were abstracted for inclusion in the study. A total of 220 participants were included, comprising 110 cases and 110 matched controls. Controls were selected from patients with drug-susceptible tuberculosis, matched by sex and age group, for each identified case of multidrug-resistant tuberculosis (MDR-TB). Eligible cases included patients with rifampicin-resistant tuberculosis, isoniazid-resistant tuberculosis, or laboratory-confirmed MDR-TB. Patients younger than 18 years were excluded from the study. Treatment outcomes for MDR-TB patients were categorized according to the World Health Organization criteria for defining treatment outcomes [9] (Annex).

**Data collection:** data were collected consecutively of DR-TB patients file records and patients with drug susceptible TB enrolled at NTH during the period of 2018-2020. This was done using a data abstraction tool adapted from previous studies [10,11] containing the following variables of the social-demographic and clinical characteristics of the study participants; sex, age, marital status, residence, family size, HIV status, interruption of TB treatment, history of previous treatment, contact with TB patients, contact with DR-TB patients, sub-optimal dosage and poor drug absorption, incorrect management of individual cases by clinician, use of anti-TB drugs of unproven quality and healthcare worker.

### Definitions

**Drug-resistant tuberculosis (DR-TB):** tuberculosis caused by *Mycobacterium tuberculosis* strains that are resistant to at least one first-line anti-TB drug.

**Multidrug-resistant tuberculosis (MDR-TB):** a form of TB that is resistant to at least both isoniazid and rifampicin, the two most powerful first-line anti-TB drugs.

**Risk factors:** characteristics, conditions, or behaviours that increase the likelihood of developing a disease or adverse health outcome.

**Case-control study:** an observational study design in which individuals with a disease or outcome of interest (cases) are compared to individuals without the outcome (controls) to identify exposures or risk factors associated with the condition.

**Treatment outcomes:** standardized measures used to assess the results of therapy in TB patients, typically including cure, treatment completion, treatment failure, death, loss to follow-up, and not evaluated. Note: all TB-related definitions were adapted from World Health Organization (WHO) TB guidelines.

**Statistical analysis:** data was cleaned using Microsoft Excel 2019, entered, processed and analyzed by IBM Statistical Package for Social Science (SPSS) version 26 software. The data had continuous and categorical variables. The mean, median and range were the statistics used to describe continuous variables while frequency and percentage distributions were used to describe the categorical variables. Both a univariable and multiple logistic regression were used to determine associated risk factors for MDR-TB and treatment outcome. Odds ratio > 1 shows positive association. Univariable analysis was performed to select variables to include in the multivariable logistics regression model at p<0.1. All the selected variables were then included in the multivariable model using enter method.

**Ethical considerations:** ethical approval for the study was obtained from the Tropical Disease Research Centre (TDRC) (TRC/C4/10/2022) a research institute based in Ndola-Zambia, and all approved data were handled with strict confidentiality. In addition, permission to conduct the study was granted by Senior Medical Superintendent at Ndola Teaching Hospital, and the Ministry of Health Copperbelt Provincial Health Office.

**Informed consent:** it was also obtained from the study participants and could withdraw from the study anytime without any consequences. The study posed minimal risk to participants, as no personal identifiers were used to ensure anonymity of records.

## Results

**General characteristics of the study population:** a total of 220 participants (110 cases and 110 controls) were selected in this study. A total enumeration was done for the cases, with a total of 110, which were paired with an equal number of controls. The mean age for the study was 36.67±9.94. As shown in Table 1, the majority were males, 156 (70.9%). The highest number of respondents was aged between 35 and 55 years. According to family size, the highest number of respondents were those in the category 1-5, with 153 (69.5%). More than half of the participants were married 122 (55.5%), followed by those who were single 78 (35.5%), and most were coming from urban residences 186 (84.5%). The majority of participants were HIV-positive, accounting for 123 (55.9%). A total of 26 (11.8%) had experienced interruption of tuberculosis treatment, while 85 (38.6%) reported a history of previous treatment. Regarding exposure, 75 (34.1%) had a positive history of contact with TB patients, and 61 (27.1%) reported contact with patients with drug-resistant TB (DR-TB).

**Table 1:** general characteristics of the study population

Description	Frequency (n)	Percentage (%)
**Age**		
Less than 35 years	87	39.5
35-55 years	123	55.9
Greater than 55 years	10	4.5
**Sex**		
Male	156	70.9
Female	64	29.1
**Family size**		
1 - 5	153	69.5
More than 5	67	30.5
**Marital status**		
Single	78	35.4
Married	122	55.4
Separated	7	3.2
Widowed	3	1.4
Divorced	10	4.5
**Patient residence**		
Rural	34	15.5
Urban	186	84.5
**HIV status**		
Positive	123	55.9
Negative	97	44.1
**Interruption of TB treatment**		
Yes	27	12.3
No	193	87.7
**History of previous treatment**		
Yes	85	38.6
No	135	61.4
**Contact with DR-TB patients**		
Yes	61	27.7
No	159	72.3

TB: tuberculosis**;** DR-TB: Drug-resistant tuberculosis

**Factors associated with outcome variables: bivariable analysis:** Table 2 below shows bivariable analysis of the occurrence of MDR-TB and social demographic characteristics. Family size and residence were associated with the occurrence of MDR-TB (p < 0.05). Other variables such as age, sex, and marital status showed no association. The history of previous treatment, contact with a TB patient, and contact with an MDR-TB patient had an association with the occurrence of MDR-TB, with p-values of <0.0001, 0.033, and 0.010 respectively. Other variables, such as HIV status, interruption of treatment, suboptimal dosage, and poor drug absorption, incorrect management of individual cases by clinicians, use of anti-TB drugs of unproven quality, and healthcare workers showed no association. A successful treatment outcome was recorded in 73.6% of the patient population, comprising 27.3% who completed treatment and 46.4% who achieved treatment success. Unsuccessful outcomes accounted for 26.4% of patients, including 9.1% with treatment failure, 0.9% who died during treatment, and 16.4% who were lost to follow-up. Among the 110 MDR-TB patients treated between 2018 and 2020, 30 (27.3%) completed treatment, 10 (9.1%) experienced treatment failure, 1 (0.9%) died, 18 (16.4%) were lost to follow-up, and 51 (46.4%) achieved treatment success. Table 3 summarizes the treatment outcomes of patients with MDR-TB.

**Table 2:** association of MDR-TB and social demographic and clinical characteristics

Description	Yes		No		P-value
	n	%	n	%	
**Age**					0.190
Less than 35 years	49	22.3	38	17.3	
35-55 years	56	25.5	67	30.5	
Greater than 55 years	5	2.3	5	2.3	
**Sex**					0.7689
Male	77	35	79	35.9	
Female	33	15	31	14.1	
**Family size**					0.028*
1-5	69	31.4	84	38.2	
More than 5	41	18.6	26	11.8	
**Marital status**					0.825
Single	41	18.6	37	16.8	
Married	59	26.8	63	28.6	
Separated	3	1.4	4	1.8	
Widowed	2	0.9	1	0.5	
Divorced	5	2.3	5	2.3	
**Patient residence**					0.025*
Rural	23	10.5	11	5	
Urban	87	39.5	99	45	
**HIV status**					0.499
Positive	59	26.8	64	29.9	
Negative	51	23.2	46	20.9	
**Interruption of TB treatment**					0.847
Yes	21	9.5	5	2.3	
No	88	40	105	47.7	
**History of previous treatment**					<0.000*
Yes	57	25.9	28	12.7	
No	53	24	82	37.3	
**Contact with TB patients**					0.033*
Yes	45	20.5	30	13.6	
No	65	29.5	80	36.4	
**Contact with MDR-TB patients**					0.010*
Yes	39	17.7	22	10	
No	71	32.3	88	40	

TB: TB: tuberculosis**;** DR-TB: Drug-resistant tuberculosis

**Table 3:** treatment outcomes of patients with MDR-TB

Description	N	%
**Treatment outcomes**		
Successful treatment outcome	81	73.6
Unsuccessful treatment outcome	29	26.4
**Treatment endpoint**		
Treatment completed	30	27.3
Treatment failed	10	9.1
Died	1	0.9
Lost to follow	18	16.4
Treatment success	51	46.4

**Univariable and multiple logistic regression analysis of MDR-TB predictors:** the relationship between study variables and the occurrence of MDR-TB was examined using logistic regression. The study variables, baseline age, sex, family size, patient residence, marital status, HIV status, interruption of TB treatment, history of previous treatment, contact with TB patient, contact with MDR-TB patient, suboptimal dosage and poor drug absorption, incorrect management of individual cases by clinicians, use of anti-TB drugs of unproven quality and healthcare worker were entered into a logistic regression model. The backward selection model was used; it removes all other variables without a statistically significant p-value. The variables that remained were family size, residence, history of previous treatment, contact with TB patient, and contact with MDR-TB patient. The multiple logistic regression model indicated a strong association between a family size of 1-5 and the occurrence of MDR-TB (AOR=1.44; 95% CI: 0.74-2.79). There was also a powerful statistical association between contact with TB patient and MDR-TB (AOR=1.67; 95% CI: 0.56-4.92). The analysis indicated that living in the rural area had a negative association with MDR-TB development (aOR, 0.42; 95% CI: 0.19 to 0.91). There was also a negative association between a history of previous treatment and developing MDR-TB (aOR=0.32; 95% CI: 0.17-0.61), as well as that between contact with an MDR-TB patient and MDR-TB development (aOR=0.50; 95% CI: 0.16-1.52) (Table 4).

**Table 4:** univariable and multiple logistic regression analysis of MDR-TB predictors

Description	Cases	Control	COR (95% CI)	AOR (95% CI)	P-value
	n(%)	n(%)			
**Family size**					
1-5	41 (18.6)	26 (11.8)	1	1	0.069
More than 5	69 (31.4)	84 (38.2)	1.920(1.069-3.448)	1.44(0.737-2.793)	0.029*
**Residence**					
Urban	87 (39.5)	99 (45.0)	1	1	0.379
Rural	23 (10.5)	11 (5.0)	0.420(0.194-0.911)	0.46(0.194-1.080)	0.028*
**History of previous treatment**					
No	53 (24.0)	82 (37.3)	1	1	<0.001*
Yes	57 (25.9)	28 (12.7)	0.318 (0.180-0.561)	0.32(0.171-0.610)	0.013*
**Contact with TB patient**					
No	65 (29.5)	80 (36.4)	1	1	0.214
Yes	45 (20.5)	30 (13.6)	0.542(0.308-0.954)	1.67(0.564-4.923)	0.034*
**Contact with MDR-TB patient**					
No	71 (32.3)	88 (40.0)	1	1	0.178
Yes	39 (17.7)	22 (10.0)	0.455(0.)	0.50(0.163-1.520)	0.011*

MDR-TB: multidrug-resistant tuberculosis; TB: tuberculosis

## Discussion

This study was conducted to determine the risk factors and treatment outcomes of drug-resistant tuberculosis among patients treated at Ndola Teaching Hospital from 2018 to 2020. A positive association was observed between a smaller family size (1-5 members) and MDR-TB occurrence, as well as between contact with a TB patient and MDR-TB. Conversely, residing in rural areas was negatively associated with MDR-TB development. Similarly, a history of previous TB treatment and contact with MDR-TB patients were both negatively associated with MDR-TB occurrence. In terms of treatment outcomes, more than two-thirds (73.6%) of MDR-TB patients achieved successful treatment, while just over a quarter (26.4%) experienced poor outcomes, including treatment failure, death, or loss to follow-up. Family size was found to be significantly associated with the occurrence of MDR-TB. This could be due to the fact that those in smaller family sizes are more likely to live in smaller, roomed apartments. The smaller the living space and the more concentrated the population within that space, the higher the chance of a TB-infected person transmitting the bacteria to others in a prolonged and close-contact situation [12,13]. These factors indirectly favor the transmission of the disease compared to those who came from families with numbers greater than 6, as they were likely to have been living in a more spacious room. These findings are in agreement with a previous study, which showed that a reduced family size was more likely to be predisposed to TB [14]. However, other studies found contrary results to our study, citing that smaller housing units had an increased risk of TB transmission [15,16]. In our current study, there was a significant association between contact with a TB patient and MDR-TB. A study in Ethiopia and Tanzania showed that contact with MDR-TB patients had almost 9 times the likelihood of MDR-TB occurrence and an increase in TB incidence in household contacts, respectively [17,18].

Further evidence in sub-Saharan Africa showed a high risk of TB in household contacts [19]. We found that individuals living in a rural area were less likely to have MDR-TB. This could possibly be explained by the relative population densities in urban areas compared to rural areas, increasing the likelihood of contact between TB-infected and TB non-infected individuals. Our findings corroborate the findings from other studies that found that urban areas had more cases of TB, including in China [20,21] and Zambia [22]. However, poor outcomes were observed in the rural areas, possibly due to inequities that exist between urban and rural areas. Contrasting results were reported in Ethiopia were MDR-TB was seen to be higher in rural areas than in urban areas, a situation possibly driven by poor adherence of TB patients to treatment, potentiated by a lack of well-established health delivery systems for TB care and management in rural areas [23,24]. There was also a negative association between a history of previous treatment and developing MDR-TB. This could be explained by the low TB treatment failure rate as seen in a previous study, where the treatment success rate for new TB smear-positive cases has greatly improved from 77% in the 2002 cohort to 88% for the 2012 cohort in Zambia [25]. The present study found a negative association between contact with MDR-TB patients and MDR-TB development. A previous study reported similar findings in Peru in 2014, where household contacts of MDR-TB patients had a lower risk of developing MDR-TB than non-household contacts. The study suggested that this might be due to household contacts being more likely to receive early detection and treatment, as well as more likely to adopt infection control measures [17]. Several other studies have, however, reported contrasting results, with contacts of MDR-TB more likely to develop MDR-TB [26-28], possibly due to differences in transmission dynamics.

The treatment outcomes in this study show that of the 110 MDR-TB patients treated from 2018 to 2020, 30 (27.3%) had completed treatment, 10 (9.1%) had treatment failure, 1 had died during the course of treatment, 18 (16.4%) had been lost to follow-up and 51 (46.4%) had treatment success. A successful treatment outcome was recorded for 73.6% of the patient population, findings that are similar to a study done in India [29]. In the case of Zambia, this could be attributed to the recent expansion of Diagnostic, Treatment, and Care (DTC) services, implementation of shorter and more effective drug regimens for resistant TB, decentralization of care closer to communities, strengthened TB/HIV integration, improved data management, and monitoring [30]. Despite this progress, Zambia continues to face challenges that contribute to unsuccessful treatment outcomes, as reflected in this study, where one-quarter of patients were lost to follow-up. Contributing factors towards loss to follow-up were highlighted by Daka *et al*. and Mishra *et al*. such as poverty, alcohol abuse, and HIV co-infection; treatment-related factors such as drug strength and high pill burden; and health system weaknesses such as poor patient-provider communication, inadequate counseling, and limited access to healthcare facilities [31,32]. These findings are consistent with a study conducted in Ethiopia, where loss to follow-up accounted for 12.8% of patients [31]. Addressing these barriers requires strengthening the health delivery system to better support TB patients and improve treatment adherence. This resonated with a previous study in Ethiopia, where loss to follow-up accounted for 12.8% of the patients [33].

**Public health implications:** these results have direct public health implications for Zambia´s End TB Strategy and some of the key global health priorities. The high proportion of unsuccessful outcomes, largely due to loss to follow-up, challenges Zambia´s goal of achieving improved treatment success rates and universal access to quality TB care. At the same time, the identified risk factors, such as household contact and HIV co-infection, show the need for integrated, patient-centered interventions that strengthen adherence and address social determinants of health. Addressing these gaps is important for Zambia to meet its national TB targets as outlined in the National Strategic Plan (NSP) for TB prevention, care and control 2022-2026 which envisions a TB-free Zambia by 2030 [34] and to contribute to the WHO End TB Strategy: to end the global tuberculosis epidemic by 2030, reduce TB incidence by 80% and TB deaths by 90% [35] and Sustainable Development Goal 3.3 [36] of ending TB as a global public health threat by 2035. The study effectively investigated MDR-TB and its potential, multiple risk factors, and with no loss to follow-up. However, dependence on existing hospital records posed a major challenge of having missing or inaccurate data, causing some sort of information bias. Recall bias was also another challenge, as patients had to remember past exposures or treatment histories. Lastly, the findings may not be fully generalizable to other regions in Zambia due to differences in healthcare settings and patient characteristics.

## Conclusion

The study found that smaller family size and contact with TB patients were risk factors for developing MDR-TB, while living in rural areas, having a history of previous TB treatment, and contact with MDR-TB patients were negatively associated with its occurrence. With males and HIV-positive individuals forming a large proportion of the study population, the findings show the importance of integrating TB and HIV programs and tailoring interventions to households and exposed groups. Although treatment outcomes were generally positive, with nearly three-quarters of MDR-TB patients achieving success, the notable proportion of unsuccessful cases underscores the need for strengthened patient-centered care, adherence support, and equitable access to healthcare across both rural and urban settings.

### What is known about this topic

- Multidrug-resistant tuberculosis is a major global health challenge with poor treatment outcomes;
- HIV co-infection increases vulnerability to tuberculosis and multidrug-resistant tuberculosis;
- Household and patient contact are recognized risk factors for tuberculosis transmission.

### What this study adds

- Smaller family size and contact with tuberculosis patients significantly increase multidrug-resistant tuberculosis risk;
- Rural residence and prior tuberculosis treatment reduce multidrug-resistant tuberculosis development likelihood;
- Nearly three-quarters of multidrug-resistant tuberculosis patients achieved successful treatment outcomes, highlighting progress but leaving room for improved adherence support.

## Annex

**Annex**: supplementary material (PDF - 265KB)
